# A precision medicine approach to sex-based differences in ideal cardiovascular health

**DOI:** 10.1038/s41598-021-93966-3

**Published:** 2021-07-21

**Authors:** Jane A. Leopold, Elliott M. Antman

**Affiliations:** Division of Cardiovascular Medicine, Brigham and Women’s Hospital, Harvard Medical School, 77 Avenue Louis Pasteur, NRB0630K, Boston, MA 02115 USA

**Keywords:** Cardiology, Medical research

## Abstract

Cardiovascular disease risk factor profiles and health behaviors are known to differ between women and men. Sex-based differences in ideal cardiovascular health were examined in the My Research Legacy study, which collected cardiovascular health and lifestyle data via Life’s Simple 7 survey and digital health devices. As the study overenrolled women (n = 1251) compared to men (n = 310), we hypothesized that heterogeneity among women would affect comparisons of ideal cardiovascular health. We identified 2 phenogroups of women in our study cohort by cluster analysis. The phenogroups differed significantly across all 7 cardiovascular health and behavior domains (all p < 0.01) with women in phenogroup 1 having a lower Life’s Simple 7 Health Score than those in phenogroup 2 (5.9 ± 1.3 vs. 7.6 ± 1.3, p < 0.01). Compared to men, women in phenogroup 1 had a higher burden of cardiovascular disease risk factors, exercised less, and had lower ideal cardiovascular health scores (p < 0.01). In contrast, women in phenogroup 2 had fewer cardiovascular risk factors but similar exercise habits and higher ideal cardiovascular health scores than men (p < 0.01). These findings suggest that heterogeneity among study participants should be examined when evaluating sex-based differences in ideal cardiovascular health.

## Introduction

With the advent of precision medicine, advanced phenotyping of patients and populations has led to the recognition that there is inherent heterogeneity among individuals at-risk for and with established cardiovascular diseases^1,2^. In population-based studies, this heterogeneity has been attributed, in part, to several factors, including age, sex, race and ethnicity, geographic region, or socioeconomic differences among participants^3^. By using an integrative approach to patient data from diverse sources, precision phenotyping has the potential to identify key determinants of heterogeneity as well as inform what constitutes cardiovascular health and disease for an individual or population.

Although substantial progress has been made in our understanding of sex-based differences in cardiovascular disease and attendant risk factors, traditional studies have not explored heterogeneity among participants and women in particular. As cardiovascular disease remains the leading cause of death for women in the United States^4,5^, recognizing heterogeneity among women achieves importance. While the prevalence of coronary heart disease is ~ 6.2% in women age ≥ 20 years, the mortality rate from cardiovascular diseases for women of all ages is 41.7%^4^. This may result from the high burden of cardiovascular disease risk factors in women that contribute to less than ideal cardiovascular health. Current estimates indicate that 68.1% of women are either overweight or obese (BMI ≥ 25 kg/m^2^), 40.4% have a total cholesterol level of ≥ 200 mg/dL, 42.8% have hypertension, 12.7% are current smokers, and 31.3% are pre-diabetic while another 11.7% have been diagnosed with diabetes mellitus^4^.

The American Heart Association has prioritized ideal cardiovascular health for all as a strategic impact goal to decrease cardiovascular mortality and increase health equity as well as overall health and well-being^6,7^. Ideal cardiovascular health has been defined by a composite of modifiable health factors (blood pressure, fasting blood sugar, cholesterol levels, healthy weight) and health behaviors (diet, exercise and activity, tobacco use)^8^. The relationship between ideal cardiovascular health and cardiovascular outcomes has been demonstrated. Several studies that have shown that ideal cardiovascular health is associated with a lower incidence of subclinical and established cardiovascular disease as well as cardiovascular mortality^9–13^. Despite this, over the past 20 years, ideal cardiovascular health has declined in community-based populations^11,13^.

Sex-based differences in ideal cardiovascular health have been examined in relatively few studies. One analysis from an eastern European cohort found that compared to men, women had a higher prevalence of ideal scores for blood pressure, healthy weight, healthy diet, and smoking, but a lower prevalence of ideal scores for activity and cholesterol levels^14^. A study from the Multi-Ethnic Study of Atherosclerosis (MESA) reported similar results, although in this cohort women had a higher average body mass index (BMI) than men^15^. In the current study, we examined sex differences in ideal cardiovascular health in the American Heart Association’s My Research Legacy study. In contrast to the aforementioned studies that collected data via in-person interview and exam, My Research Legacy was a direct-to-participant study that aimed to understand ideal cardiovascular health and the role of digital health data in this assessment. Since the study enrolled a disproportionate number of women, we hypothesized that there was heterogeneity among women in the study and that this heterogeneity would impact the outcome of sex-based comparisons in cardiovascular health and behaviors.

## Methods

### Study design and data acquisition

My Research Legacy was a direct-to-participant study sponsored by the American Heart Association and was conducted entirely online (clinicaltrials.gov: NCT02958098)^16^. Participants self-reported cardiovascular health data. They were also given the option to register digital health devices to report weight and exercise activity data. The study was approved by the central IRB, Advarra Institutional Review Board (www.advarra.com) (Protocol #31995) and the research was performed in accordance with the Declaration of Helsinki. Participants were eligible if they had internet access, lived in the United States, and were $\ge$ 18 years old. Participants provided informed consent online and could withdraw from the study at any time. The study was conducted from November 2016 to October 2018^16^.

At study entry, participants self-reported baseline demographic information, prior history of cardiovascular diseases and completed Life’s Simple 7 survey questions^6,7,16^. Socioeconomic data was derived from the National Neighborhood Data Archive (https:openicpsr.org) based on zip code tabulation areas. The dataset includes an affluence index variable that was constructed from the US Census Bureau’s American Community Survey. The affluence index factored in concentrations of adults with a college education, incomes > $75 K, and employment in managerial and professional occupations for individual zip code tabulation areas. The index was defined as an average of these census indicators, ranged from 0 to 1.0, and has been validated^17^. The dataset covering the years 2013–2017 was selected for use and a zip code tabulation area to zip code crosswalk was used to align the affluence index data with participant zip codes^18^.

Individuals that registered a digital device received a unique link to Validic (Validic Inc., Durham, NC) to transmit data. Weight data were recorded by a smart scale. In order to compare exercise and activity data to self-reported survey data, exercise and activity data as well as daily step count were recorded for 7 consecutive days. Self-reported data were uploaded to Amazon Web Services secure servers and deidentified using a platform developed and managed by The Broad Institute (Cambridge, MA) and REAN Cloud LLC (Herndon, VA)^16^.

### Analytic approach

Participant responses to Life’s Simple 7 categories of health factors and behaviors was combined with self-reported demographic, cardiovascular risk factor and health history data, and digital health device data to form the dataset. Heterogeneity was examined by first transforming some categorical variables to numeric binary variables and a correlation matrix based on Pearson correlation coefficients was established. Variables with a correlation coefficient > 0.7 were evaluated and the results were filtered to eliminate redundancy. This decreased the number of variables from 31 to 26^19^. Agglomerative hierarchical clustering was then utilized to group participants and phenotypic variables. Variables were scaled to facilitate comparison and clustering was performed based on Ward’s method and squared Euclidean distance using hclust in R (version 3.6.2)^20^. Data were visualized by heatmap using heatmap.2 in R (version 3.6.2).

In order to resolve clusters and explore similarity between participants, a factor analysis of mixed data was performed. Factor analysis of mixed data was used to reduce the number of variables to the smallest number while maintaining maximal information and to identify proximity between observations^21^. Dissimilarity between participants was calculated using Gower distance^22^. When calculating the Gower distance, binary categorical variables were identified as asymmetric and ordinal variables were labeled as such. The optimal number of clusters was determined by silhouette width. Clusters were identified using a partitioning around mediods algorithm. Cluster assignment was then used to identify phenogroups, which were visualized using t-distributed stochastic neighborhood embedding. Cluster analysis and visualization was performed using R (version 3.6.2) and the FactoMineR, factoextra, and Rtsne packages.

Normality of the data was tested using the Shapiro–Wilk test. Comparisons between categorical variables were performed using the chi-square test or Fisher’s exact test as appropriate. Comparisons between continuous variables were performed using t-tests or paired t-tests. Nonparametric testing was done using the Wilcoxon-rank sum test or Wilcoxon matched pairs signed rank test. Multivariable ordinal logistic regression was performed using 4 age group strata (18–34, 35–49, 50–64, and ≥ 65 years); 2 race and ethnicity strata (white and non-white); and 4 affluence index strata (0– < 0.31, 0.31– < 0.41, 0.41– < 0.53, ≥ 0.53). Collinearity was examined using pairwise correlation comparisons. To compare women in each of the clusters to men with similar characteristics, propensity score matching using a logit model was done adjusting for age, race and ethnicity, region, and affluence index at a ratio of 2:1 (women:men). Data are presented as mean $\pm$ SD. P values < 0.05 were considered statistically significant. Data were analyzed using Stata 15/SE 15.1 (StataCorp LLC, College Station, TX) and Prism 9.0 (GraphPad, San Diego, CA).

## Results

### Differences in self-reported data between women and men enrolled in My Research Legacy

The My Research Legacy study enrolled 1,561 participants: 1,251 women and 310 men. The study enrolled individuals across all age groups, from each of the 50 states, and across all socioeconomic strata as determined by affluence index, indicating that our sample was generalizable to community-based populations in the United States (Suppl. Figure 1). Women were younger than men (43.7 ± 12.5 vs. 46.3 ± 15.0 years, p < 0.01) and there were differences in race and ethnicity between the groups (p < 0.03). Women also had a lower affluence index than men (p < 0.02). Women were less likely to self-report a history of cardiovascular disease than men and had less hypertension (p < 0.01). Women had a similar prevalence of diabetes mellitus and hypercholesterolemia as men, although women were less likely to be treated for hypercholesterolemia (p < 0.01). While there was no difference in BMI between women and men, women had lower systolic and diastolic blood pressures and blood glucose levels than men, but higher levels of cholesterol (p < 0.01) (Table 1). Although women reported a higher daily intake of fruit (p < 0.04) and vegetables than men (p < 0.02), they consumed fewer servings of fish per week (p < 0.01). There was no difference between women and men with respect to daily whole grain consumption, sugar-sweetened beverages per week, or other dietary habits, including avoidance of pre-packaged foods, eating out, and added salt. Women and men reported a similar number of weekly minutes of moderate exercise, but women reported fewer minutes of vigorous exercise per week than men (61.4 ± 111.1 vs. 91.3 ± 135.1 min/week, p < 0.01) (Table 2).

**Table 1:** Participant self-reported demographics and cardiovascular health.

	Entire cohort (n = 1561)	Self-reported in Life's Simple 7 survey		P value
		Women (n = 1251)	Men (n = 310)	
Age (years)	44.2 $\pm$ 13.0	43.7 $\pm$ 12.5	46.3 $\pm$ 15.0	< 0.01
**Race and ethnicity (no.)**				< 0.03
Asian	42	25	17	
Black	60	49	11	
Hispanic	68	54	14	
White	1337	1077	260	
Other	54	46	8	
**Region (no.)**				0.34
Northeast	227	190	37	
South	622	487	135	
Midwest	378	303	75	
West	334	271	63	
Affluence index	0.42 ± 0.15	0.42 ± 0.15	0.44 ± 0.16	< 0.02
Diagnosed with Cardiovascular Disease (%)	36.3	35.1	41.0	0.05
Diabetes mellitus (%)	10.3	10.2	10.7	0.83
Hypertension (%)	49.8	47.2	60.3	< 0.01
Hypercholesterolemia (%)	53.4	53.1	54.8	0.58
**Medications (%)**				
Diabetes mellitus	8.8	8.8	8.7	0.96
Hypertension	32.5	30.5	40.3	< 0.01
Hypercholesterolemia	20.6	18.2	30.0	< 0.01
**Smoking status (%)**				0.58
Current	6.9	7.4	5.2	
Quit < 12 months	3.8	3.8	3.6	
Quit ≥ 12 months	23.6	23.4	24.5	
Never	65.7	65.4	66.8	
Weight (kg)	84.2 $\pm$ 24.4	81.6 $\pm$ 23.9	94.6 $\pm$ 23.8	< 0.01
Height (cm)	167.6 $\pm$ 9.6	164.8 $\pm$ 7.3	179.0 $\pm$ 9.3	< 0.01
BMI (kg/m^2^)	29.9 $\pm$ 8.2	30.1 $\pm$ 8.6	29.4 $\pm$ 6.5	0.19
Systolic blood pressure (mmHg)*	118.1 $\pm$ 12.7	117.1 $\pm$ 12.8	122.2 $\pm$ 11.9	< 0.01
Diastolic blood pressure (mmHg)*	73.4 $\pm$ 8.7	72.9 $\pm$ 8.7	75.2 $\pm$ 8.1	< 0.01
Total cholesterol (mg/dL)*	188.7 $\pm$ 29.2	190.5 $\pm$ 27.7	181.4 $\pm$ 33.7	< 0.01
Blood glucose (mg/dL)*	99.3 $\pm$ 18.1	98.2 $\pm$ 18.2	103.7 $\pm$ 17.3	< 0.01

*Contains data imputed from Life’s Simple 7.

Categorical variables are analyzed by Chi-Square test.

Continuous variables are analyzed by t-test.

Non-parametric variables were analyzed by Wilcoxon rank-sum test.

**Table 2:** Participant self-reported diet and weekly exercise data.

	Entire cohort (n = 1561)	Self-reported in Life's Simple 7 survey		P value
		Women (n = 1,251)	Men (n = 310)	
**Diet**				
Vegetables/day (cups)	1.9 $\pm$ 1.3	1.9 $\pm$ 1.3	1.7 $\pm$ 1.2	< 0.02
Fruit/day (cups)	1.4 $\pm$ 1.1	1.4 $\pm$ 1.1	1.2 $\pm$ 1.1	< 0.04
Fish (servings/week)	0.9 $\pm$ 1.0	0.9 $\pm$ 1.0	1.1 $\pm$ 1.1	< 0.01
Whole grains (servings/day)	1.6 $\pm$ 1.2	1.6 $\pm$ 1.2	1.6 $\pm$ 1.2	0.45
Sugary drinks (servings/week)	2.4 $\pm$ 3.4	2.4 $\pm$ 3.4	2.4 $\pm$ 3.3	0.90
Avoid prepackaged foods (%)	52.2	52.2	52.3	0.99
Avoid eating out (%)	37.6	38.3	34.8	0.26
Avoid salt at home (%)	56.6	56.6	56.8	0.95
**Exercise**				
Moderate exercise (min/week)	204.0 $\pm$ 215.0	201.4 $\pm$ 215.7	214.4 $\pm$ 212.4	0.34
Vigorous exercise (min/week)	67.3 $\pm$ 116.8	61.4 $\pm$ 111.1	91.3 $\pm$ 135.1	< 0.01

Categorical variables are analyzed by Chi-Square test.

Continuous variables are analyzed by t-test.

**Figure 1:**
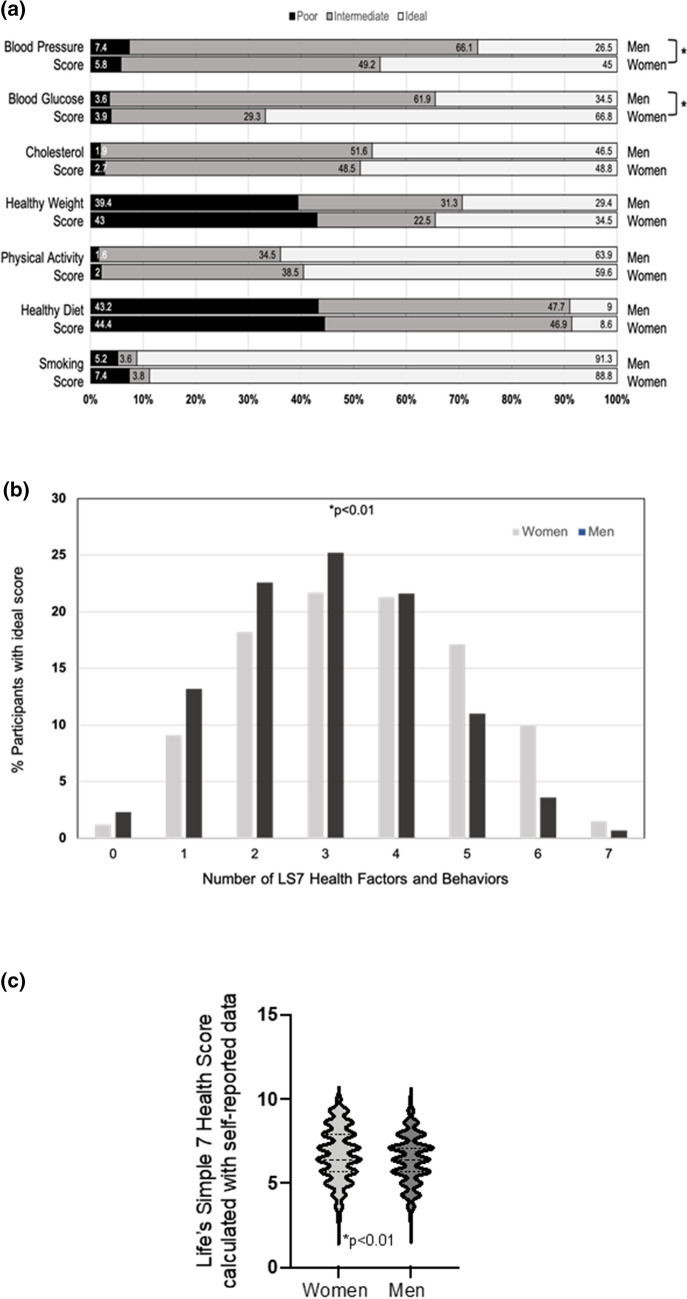
Gender differences in Life’s Simple 7 health factors and behaviors. (**a**) The percentage of poor, intermediate, and ideal scores for women (n = 1251) and men (n = 310) for each of the 7 health factors and behaviors assessed in Life’s Simple 7. *p < 0.01. (**b**) The number of Life’s Simple 7 health and behavior categories for which participants achieved an ideal score. Data are shown for women and men. (**c**) Violin plots of the Life’s Simple 7 Health Score calculated using self-reported data for women compared to men. Median and quartiles are represented by dashed lines. *p < 0.01.

Life’s Simple 7 utilizes these data to categorize cardiovascular health and behaviors as poor, intermediate, or ideal based on a set of predefined criteria^6–8^. Women and men had a similar distribution of participants that had poor, intermediate, or ideal scores for Life’s Simple 7 smoking status, physical activity, healthy weight, healthy diet, and cholesterol scores. There were, however, significant differences between men and women with respect to blood pressure and blood glucose scores (p < 0.01) (Fig. 1a). There was also a significant difference in the number of women who met ≥ 5 criteria for ideal cardiovascular health compared to men (p < 0.01)(Fig. 1b). Taken together, it is not surprising that women had a higher Life’s Simple 7 Health Score than men (6.7 ± 1.6 vs. 6.4 vs. 1.4, p < 0.01) (Fig. 1c). After adjusting for age, race and ethnicity, region and affluence index, the odds of having an ideal cardiovascular health score remained higher for women compared to men (OR 1.4 95% CI 1.1–1.7, p < 0.01) (Suppl. Figure 2).

### Two phenogroups of women identified in the study cohort

**Figure 2:**
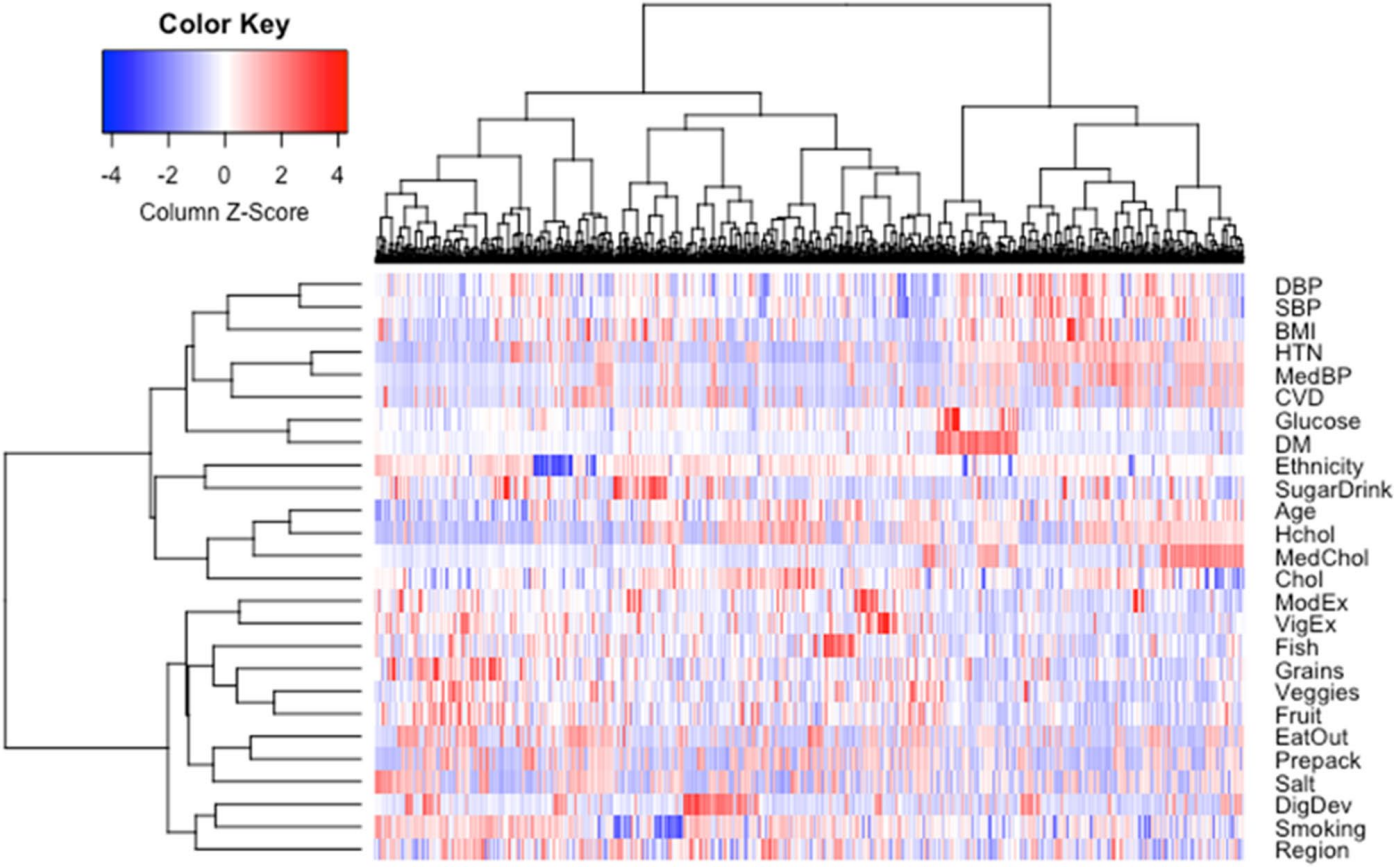
Heterogeneity among women. Hierarchical clustering of continuous and categorical variables derived from Life’s Simple 7 survey responses was performed to create a phenotype heatmap for women (n = 1,251) enrolled in the study. The heatmap reveals heterogeneity among women. DBP, diastolic blood pressure; SBP, systolic blood pressure; BMI, body mass index; HTN, hypertension; MedBP, takes medications for blood pressure; CVD, prior history of prior cardiovascular disease; DM, diabetes mellitus; SugarDrink, number of sugar-sweetened beverages; Hchol, hypercholesterolemia; MedChol, takes medications for cholesterol; Chol, total cholesterol; ModEx, weekly minutes of moderate exercise; VigEx, weekly minutes of vigorous exercise; Fish, servings of fish per week; Grains, servings of whole grains per day; Veggies, cups of vegetables per day; Fruit, cups of fruit per day; EatOut, avoid eating out; Prepack, avoid prepackaged foods; Salt, avoid added salt; DigDev, registered a digital health device; Smoking, smoking status; Region, region of the United States.

**Figure 3:**
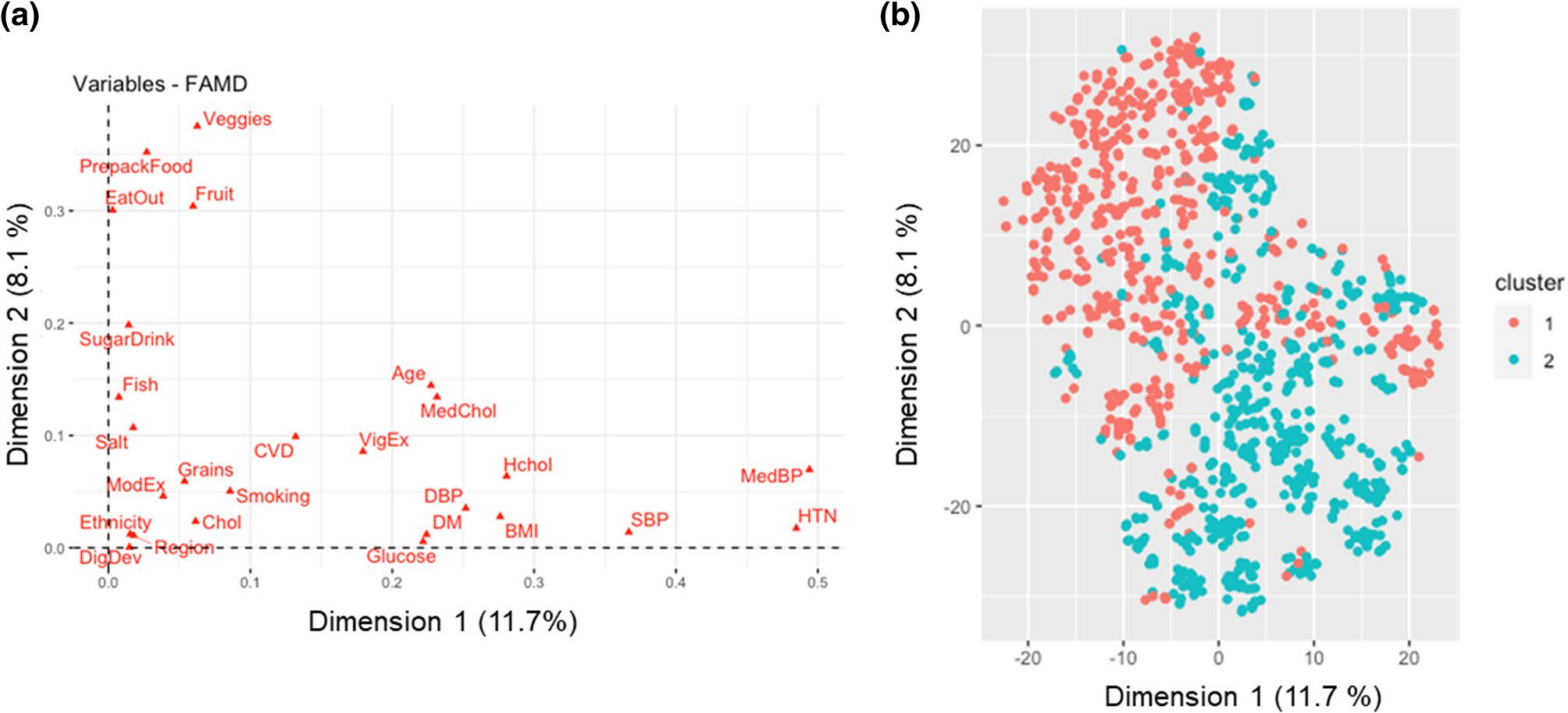
Two phenogroups of women in My Research Legacy. (**a**) A factor analysis of mixed data using variables from Life’s Simple 7 health and behavior categories demonstrated which variables aligned with the first and second dimensions to explain 19.8% of the variability among women. Veggies, cups of vegetables per day; PrepackFood, avoid prepackaged foods; EatOut, avoid eating out; Fruit, cups of fruit per day; SugarDrink, number of sugar-sweetened beverages; Fish, servings of fish per week; Salt, avoid added salt; CVD, prior history of cardiovascular disease; MedChol, cholesterol medications; Grains, servings of whole grains per day; VigEx, weekly minutes of vigorous exercise per week. Ethnicity, race and ethnicity; Region, region of the United States; DigDev, registered a digital health device; Chol, total cholesterol; ModEx, weekly minutes of moderate exercise; DBP, diastolic blood pressure; Hchol, hypercholesterolemia; MedDM, takes medication for diabetes mellitus; Glucose, blood glucose levels; SBP, systolic blood pressure; DM, diabetes mellitus; BMI, body mass index; HTN, hypertension; MedBP, takes medications for blood pressure. (**b**) Cluster analysis based on partitioning around mediods revealed 2 clusters of women in the study.

As the study included a large sample of women, we sought to determine if there was heterogeneity among women and if this would influence the advantageous cardiovascular health profile observed when we compared women to men. To assess this, we first performed hierarchical clustering and created a phenomap (phenotype heat map^19^) for women enrolled in the study. This revealed that there was heterogeneity seen among women despite small groups of individuals sharing common characteristics (Fig. 2). As a result of this observed phenotypic heterogeneity, we hypothesized that there were clusters or phenogroups of women in the study. Next, we performed a factor analysis of mixed data to understand contributors to the heterogeneity among women. This demonstrated that 19.8% of the variability was explained by the first two dimensions, which focused on cardiovascular health profile and dietary variables, respectively (Fig. 3a, Suppl. Figure 3). Using silhouette width, we determined that the optimal number of clusters was 2 and utilized a partitioning around mediods algorithm to identify the clusters (Fig. 3b, Suppl. Figure 4). This analysis assigned 614 women to cluster 1 and 637 women to cluster 2.

The two phenogroups of women had different cardiovascular health and behavior profiles with women in cluster 1 (n = 614) representing a higher risk cardiovascular phenotype as compared to women in cluster 2 (n = 637). Women in cluster 1 were older (p < 0.01), more likely to live in the South (p < 0.01) and have a lower affluence index (p < 0.01) but there was no difference in race and ethnicity between the phenogroups. Women in cluster 1 also had a higher prevalence of cardiovascular diseases, diabetes mellitus, hypertension, and hypercholesterolemia than women in cluster 2 and were more likely to have used tobacco (all p < 0.01). Women in cluster 1 reported higher weight and BMI, systolic and diastolic blood pressures, and blood glucose and cholesterol levels (all p < 0.01). There were also differences in diet and dietary habits between the phenogroups with women in cluster 1 reporting lower consumption of vegetables, fruits, whole grains, and fish than women in cluster 2, but increased consumption of sugar-sweetened beverages (all p < 0.01). Women in cluster 1 were less likely to avoid pre-packaged foods and eating out than women in cluster 2 (p < 0.01), but more likely to avoid salt at home (all p < 0.04). Exercise profiles were substantially different between women in the clusters with women in cluster 1 completing fewer weekly minutes of moderate exercise (179.8 ± 204.5 vs. 222.2 ± 224.1 min/week, p < 0.01) and vigorous exercise (36.3 ± 82.0 vs. 85.5 ± 128.8 min/week, p < 0.01) than women in cluster 2 (Table 3). There were significant differences between cluster 1 and 2 with respect to the distribution of women that were categorized as poor, intermediate, and ideal for the smoking, physical activity, healthy diet, healthy weight, blood glucose, cholesterol, and blood pressure cardiovascular health and behavior categories with women in cluster 1 less likely to achieve an ideal score compared to women in cluster 2. This resulted in women in cluster 1 having significantly lower Life’s Simple 7 Health Scores than women in cluster 2 (5.9 ± 1.3 vs 7.6 ± 1.3, p < 0.01) (Table 4). After adjusting for age, race and ethnicity, region and affluence index, the odds of having an ideal cardiovascular health score remained higher for women in cluster 2 compared to those in cluster 1 (OR 7.1 95% CI 5.7–8.9, p < 0.01).

**Table 3:** Phenogroup self-reported Life's Simple 7 data.

	Reported in Life's Simple 7 survey		
	Cluster 1 (n = 614)	Cluster 2 (n = 637)	P value
Age (years)	47.4 $\pm$ 10.9	40.0 $\pm$ 12.8	< 0.01
**Region (no.)**			< 0.01
Northeast	98	92	
South	273	214	
Midwest	145	158	
West	98	173	
Affluence index	0.40 ± 0.14	0.43 ± 0.15	< 0.01
Diagnosed with Cardiovascular Disease (%)	54.2	16.6	< 0.01
Diabetes mellitus (%)	16.3	4.4	< 0.01
Hypertension (%)	73.1	22.3	< 0.01
Hypercholesterolemia (%)	78.2	28.9	< 0.01
**Medications (%)**			
Diabetes mellitus	14.2	3.6	< 0.01
Hypertension	50.2	11.6	< 0.01
Hypercholesterolemia	31.9	5.0	< 0.01
**Smoking status (%)**			< 0.01
Current	8.6	6.1	
Quit < 12 months	4.7	3.0	
Quit ≥ 12 months	28.7	18.4	
Never	58.0	72.5	
**Health data**			
Weight (kg)	87.7 $\pm$ 24.4	75.8 $\pm$ 21.8	< 0.01
Height (cm)	164.4 $\pm$ 7.5	165.1 $\pm$ 7.1	0.11
BMI (kg/m^2^)	32.5 $\pm$ 9.0	27.8 $\pm$ 7.5	< 0.01
Systolic blood pressure (mmHg)*	121.1 $\pm$ 14.0	113.2 $\pm$ 10.1	< 0.01
Diastolic blood pressure (mmHg)*	75.4 $\pm$ 9.4	70.4 $\pm$ 7.2	< 0.01
Total cholesterol (mg/dL)*	196.1 $\pm$ 29.0	185.0 $\pm$ 25.4	< 0.01
Blood glucose (mg/dL)*	101.5 $\pm$ 19.7	94.9 $\pm$ 15.9	< 0.01
**Diet and exercise data**			
Vegetables/day (cups)	1.6 $\pm$ 1.2	2.2 $\pm$ 1.4	< 0.01
Fruit/day (cups)	1.2 $\pm$ 0.9	1.6 $\pm$ 1.2	< 0.01
Fish (servings/week)	0.8 $\pm$ 1.0	1.0 $\pm$ 1.0	< 0.01
Whole grains (servings/day)	1.4 $\pm$ 1.1	1.7 $\pm$ 1.2	< 0.01
Sugar-sweetened beverages (servings/week)	2.7 $\pm$ 3.6	2.2 $\pm$ 3.2	< 0.02
Avoid prepackaged foods (%)	33.9	69.9	< 0.01
Avoid eating out (%)	23.8	52.3	< 0.01
Avoid salt at home (%)	59.6	53.7	< 0.04
Moderate exercise (min/week)	179.8 $\pm$ 204.5	222.2 $\pm$ 224.1	< 0.01
Vigorous exercise (min/week)	36.3 $\pm$ 82.0	85.5 $\pm$ 128.8	< 0.01

*Contains data imputed from Life’s Simple 7.

Categorical variables are analyzed by Chi-Square test.

Continuous variables are analyzed by t-test.

Non-parametric variables were analyzed by Wilcoxon rank-sum test.

**Table 4:** Phenogroup Life's Simple 7 Health Factors and Behaviors Score.

	Reported in Life's Simple 7 survey		
	Cluster 1 (n = 614)	Cluster 2 (n = 637)	P value
**Life’s Simple 7 scores**			
**Smoking status score (%)**			< 0.02
Poor	8.6	6.1	
Intermediate	4.7	3.0	
Ideal	86.6	90.9	
**Physical activity score (%)**			< 0.01
Poor	2.8	1.3	
Intermediate	47.1	30.1	
Ideal	50.1	68.6	
**Healthy diet score (%)**			< 0.01
Poor	57.3	32.0	
Intermediate	37.6	55.9	
Ideal	5.1	12.1	
**Healthy weight score (%)**			< 0.01
Poor	55.4	32.1	
Intermediate	22.3	22.6	
Ideal	22.3	46.3	
**Blood glucose score (%)**			< 0.01
Poor	5.9	2.1	
Intermediate	38.1	20.7	
Ideal	56.0	77.2	
**Cholesterol score (%)**			< 0.01
Poor	3.9	1.6	
Intermediate	73.1	26.5	
Ideal	23.0	71.9	
**Blood pressure score (%)**			< 0.01
Poor	9.8	2.0	
Intermediate	68.2	30.8	
Ideal	22.0	67.2	
Health Score	5.9 $\pm$ 1.3	7.6 $\pm$ 1.3	< 0.01

Non-parametric variables were analyzed by Wilcoxon rank-sum test.

### Ideal cardiovascular health in phenogroups of women compared to men

In comparison to men (n = 310), women in the cluster 1 phenogroup were similar in age but enrolled fewer individuals who self-categorized as Asian (p < 0.02). Women in this phenogroup were more likely to be current smokers (p < 0.05), have diabetes (p < 0.03), hypertension (p < 0.01), hypercholesterolemia (p < 0.01), and a prior history of cardiovascular diseases (p < 0.01) than men. Women had a higher BMI (32.5 ± 9.0 vs. 29.4 ± 6.5 kg/m^2^, p < 0.01) and higher cholesterol level (p < 0.01) than men, but no difference in systolic and diastolic blood pressures or fasting blood glucose levels. There were few dietary differences between women and men, although women consumed fewer servings of fish per week and whole grains than men and were less likely to avoid prepackaged foods and eating out (all p < 0.02). Women self-reported fewer weekly minutes of moderate exercise (179.8 ± 204.5 vs. 214.4 ± 212.4 min/week, p < 0.02) and vigorous exercise (36.3 ± 82.0 vs. 135.1 ± 76.2 min/week, p < 0.01) compared to men. Thus, despite being similar age as men, women in this phenogroup had a higher burden of cardiovascular disease risk factors and lower indices of ideal cardiovascular health, which was reflected by a lower Life’s Simple 7 Health Score than men (5.9 ± 1.3 vs. 6.4 ± 1.4, p < 0.01) (Suppl. Table 1). The odds of an ideal cardiovascular health score remained lower for women in cluster 1 compared to men (OR 0.5 95% CI 0.4–0.7, p < 0.01) after adjusting for age, race and ethnicity, region, and affluence index. Next, propensity score matching was performed considering age, race and ethnicity, region and affluence index to compare women in cluster 1 with a matched sample of men. After propensity score matching, the average effect of female sex on the Health Score was –0.4 (95% CI –0.6 – –0.2, p < 0.01), indicating that female sex was associated with an average Health Score that was 0.4 points lower than that for a matched group of men, similar to what was observed in comparison to the entire cohort of men.

In contrast, women in the cluster 2 phenogroup were younger than men (40.0 ± 12.8 vs. 46.3 ± 15.0 years, p < 0.01) but there were no differences in race and ethnicity or affluence index between the groups. While there was no difference in smoking status between women and men, women had a lower prevalence of diabetes mellitus, hypertension, hypercholesterolemia, and prior history of cardiovascular diseases than men (all p < 0.01). Women in this phenogroup also had lower BMIs (27.8 ± 97.5 vs. 29.4 ± 6.5 kg/m^2^, p < 0.01), systolic and diastolic blood pressure, and fasting blood glucose levels than men (p < 0.01), but there was no difference in cholesterol levels between the groups. Women reported higher consumption of fruits and vegetables, but lower consumption of fish than men (all p < 0.01). They were also more likely to avoid prepackaged foods and eating out compared to men (p < 0.01). Interestingly, there was no difference between women and men with respect to weekly minutes of moderate or vigorous exercise. Compared to men, women in this phenogroup were more likely to have an ideal score in ≥ 5 cardiovascular health and behavior categories, which contributed to their higher Life’s Simple 7 Health Scores (7.6 ± 1.3 vs. 6.4 ± 1.4, p < 0.01) (Suppl. Table 1). The odds of an ideal cardiovascular health score remained higher for women in cluster 2 compared to men (OR 3.7 95% CI 2.8–4.8, p < 0.01) after adjusting for age, race and ethnicity, region, and affluence index. Women in cluster 2 were also compared to a matched sample of men using propensity score matching that considered age, race and ethnicity, region and affluence index in order minimize bias. After propensity score matching, the average effect of female sex on the Health Score was a 1.0 (95% CI 0.8–1.2, p < 0.01) indicating that female sex was associated with a Health Score that was 1.0 point higher than that for a matched group of men, also similar to what was observed when women in cluster 2 were compared to the entire cohort of men.

### Incorporating digital health data into Life’s Simple 7 Health Score

We next sought to determine how digital health device data informed sex-based differences in ideal cardiovascular health. Of the 390 individuals who registered digital health devices in the study, 307 were women and 83 were men. A total of 98 participants (72 women and 26 men) did not transmit digital weight data and 35 (26 women and 9 men) did not transmit digital exercise data.

A total of 132 women from cluster 1 and 103 women from cluster 2 contributed digital health data (p < 0.01). Similar to self-reported data, there were significant differences between the phenogroups with respect to digital health device-measured weight (83.2 ± 22.1 vs. 73.9 ± 18.1 kg, p < 0.01) and BMI (30.4 ± 7.8 vs. 27.0 ± 6.4 kg/m^2^, p < 0.01). When self-reported weight data were compared to digital health device-measured weight data, women in cluster 1 overreported their weight and women in cluster 2 underreported their weight (0.1 ± 4.7 vs. − 1.2 ± 4.2 kg, p < 0.04) resulting in over- and underreporting their BMI (0.0 ± 1.7 vs. − 0.4 ± 1.6 kg/m^2^, p < 0.04). This led to a reclassification of the weight score for 16 women in cluster 1 and 11 women in cluster 2 resulting in a significant difference in the distribution of women with poor, intermediate, and ideal weight scores between the phenogroups (p < 0.01) (Suppl. Table 2).

We also examined digital health device-recorded activity over a one week time period. There was no difference in the weekly minutes of moderate exercise (118.6 ± 145.1 vs. 139.6 ± 185.1 min/week, p = 0.29) or vigorous exercise (137.3 ± 209.6 vs. 159.7 ± 196.4 min/week, p = 0.36) between women in cluster 1 (n = 144) and cluster 2 (n = 137). We did, however, find that women in both phenogroups overreported their weekly minutes of moderate activity and underreported their minutes of vigorous activity. Women in cluster 1 overreported their weekly minutes of moderate activity (194. 0 ± 189.3 vs. 118.6 ± 12.1 min/week, p < 0.01) and underreported their weekly minutes of vigorous activity (42.0 ± 72.2 vs. 137.3 ± 209.6 min/week, p < 0.01). Similarly, women in cluster 2 overreported their weekly minutes of moderate activity (226.3 ± 229.9 vs. 139.6 ± 185.1 min/week, p < 0.01) and underreported their weekly minutes of vigorous activity (110.9 ± 143.9 vs. 159.7 ± 196.4 min/week, p < 0.01). Importantly, women in cluster 1 significantly underestimated their weekly minutes of vigorous activity compared to women in cluster 2 (− 95.4 ± 200.5 vs. − 48.8 ± 194.2 min/week, p < 0.05). Using digital health device-measured activity data, 106 women had reclassification of their activity score. Based on these data, there was no difference in the distribution of poor, intermediate, or ideal scores between the phenogroups. When digital health device-measured weight and activity data were incorporated into the Life’s Simple 7 Health Score, women in both phenogroups had improved their Health Score, but it remained lower for women in cluster 1 as compared to women in cluster 2 (6.4 ± 1.2 vs. 7.9 ± 1.1, p < 0.01) (Suppl. Table 2). After adjusting for age, race and ethnicity, region, and affluence index, the odds of an ideal cardiovascular health score remained higher for women in cluster 2 compared to women in cluster 1 (OR 1.9 95% CI 1.4–2.5, p < 0.01).

**Figure 4:**
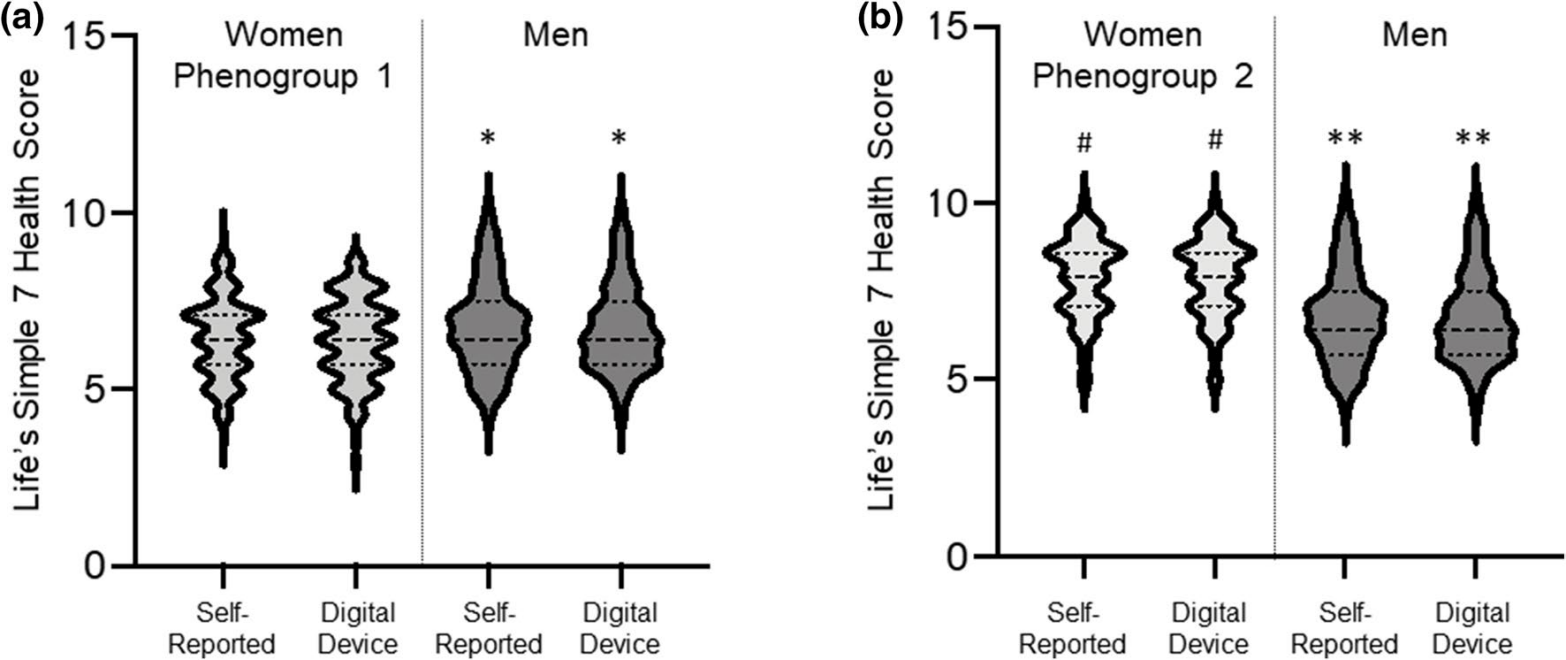
Life’s Simple 7 Health Score calculated using digital health device data. Violin plots of Life’s Simple 7 Health Score calculated using self-reported data and digital health data for individuals that had both weight and activity data available from digital health devices. (**a**) Comparison of women in phenogroup 1 (n = 122) to men (n = 53), and (**b**) Women in phenogroup 2 (n = 97) compared to men (n = 53). *p < 0.01 vs. women in phenogroup 1, self-reported or digital device data; ^#^p < 0.01 vs. women in phenogroup 1, self-reported or digital device data **p < 0.01 vs. women in phenogroup 2, self-reported or digital device data.

Next, we evaluated how use of digital health device data affected sex-based comparisons between each of the phenogroups of women and men. Women in cluster 1 were more likely than men to provide digital health device-measured weight data (p < 0.01). While there was no difference in digital health device measured weight or the difference between self-reported and digital health device measured weight between women in cluster 1 and men (n = 57), digital device calculated BMI remained higher in women than men (30.4 ± 7.8 vs. 27.4 ± 4.9 kg/m^2^, p < 0.01) as did the distribution of individuals with poor, intermediate, and ideal weight scores with women having a higher percentage of individuals categorized as poor (p < 0.02). Women in cluster 1 and men (n = 74) were equally likely to contribute exercise data. In contrast to self-reported data, there was no difference in weekly minutes of digital device-measured moderate activity; however, men did record more weekly minutes of vigorous activity than women (226.1 ± 303.0 vs. 137.3 ± 209.6 min/week, p < 0.02). There was no difference in the percent of women and men that had reclassification of their activity score on the basis of digital health device recorded data, but men had a higher percentage of individuals with an ideal activity score (67.6 vs. 53.5%, p < 0.05). When device-measured weight and activity data was incorporated into the Health Score, there was no difference between women from this phenogroup and men (6.4 ± 1.2 vs. 6.8 ± 1.2, p = 0.19) (Fig. 4) (Suppl. Table 2). There was also no significant difference in the odds for ideal cardiovascular health after adjusting for age, race and ethnicity, region, and affluence index or when comparing propensity-matched men with women from cluster 1.

Women in cluster 2 were equally likely as men to provide weight and exercise data from digital health devices. While women in this phenogroup recorded lower digital health device measured weight than men (73.9 ± 18.1 vs. 88.2 ± 18.2, p < 0.01), there was no difference in digital device calculated BMI, the difference between self-reported and measured weight, reclassification of the weight score, or the distribution of poor, intermediate, and ideal weight scores between women and men. Similar to self-reported data, there was no difference between women and men with respect to digital device-measured minutes of moderate exercise, but there was a trend towards men recording more weekly minutes of vigorous activity than women (226.1 ± 303.0 vs. 159.7 ± 196.4 min/week, p < 0.06). There was no difference in activity score reclassification or the distribution of individuals with poor, intermediate, or ideal scores between men and women. When digital health device data was utilized to calculate the Health Score, women in this phenogroup had a significantly higher score than men (7.9 ± 1.1 vs. 6.8 ± 1.2, p < 0.01) (Fig. 4) (Suppl. Table 2). After adjusting for age, race and ethnicity, region, and affluence index, the odds for having an ideal cardiovascular Health Score were higher in cluster 2 women than men (OR 1.5 95%CI 0.9–2.2, p < 0.01) and after propensity score matching, the average increase in Health Score for women in cluster 2 was 1.0 point (95% CI 0.5–1.5, p < 0.01) compared to men.

## Discussion

In this analysis of sex-based differences in ideal cardiovascular health, we found that while women were more likely than men to achieve ideal scores in ≥ 5 Life’s Simple 7 cardiovascular health and behavior categories, there was significant heterogeneity among women enrolled in My Research Legacy. A factor analysis of mixed data found cardiovascular disease risk factors and diet as determinants of variability among women and a cluster analysis identified two phenogroups of women. These two phenogroups were significantly different for each of the cardiovascular health and behavior categories with one group (cluster 1) having a higher cardiovascular disease risk profile. When compared to men enrolled in the study, women in this phenogroup were of similar age, yet had a higher prevalence of cardiovascular disease risk factors and lower overall Health Scores. In contrast, women in the other phenogroup (cluster 2) had better indices of cardiovascular health and behaviors than men and higher Health Scores. In a subset of participants, we also examined the effect of using digital health device recorded data in place of self-reported data to evaluate ideal cardiovascular health. Here, we found that women in both phenogroups under- and overreported weight and weekly minutes of exercise compared to what was recorded by the digital health device. For women in the higher risk phenogroup, substituting digital health device weight and activity data increased their Health Score such that there was no longer a significant difference in the Health Score between women and men. In contrast, for women in the lower risk phenogroup, the use of digital health device data improved their Health Score and it continued to remain significantly higher than that for men.

Prior studies have found sex differences in ideal cardiovascular health and behavior metrics^14,15,23^. The Heart Strategies Concentrating on Risk Evaluation (Heart SCORE) used baseline visit data collected between 2001 and 2004 to examine ideal cardiovascular health in a community-based study conducted in Allegheny County, PA. In this sample, there were sex-based differences for smoking status, BMI, blood pressure, blood glucose, physical activity, and total cholesterol with women having better health status in all areas except physical activity and total cholesterol^24,25^. The MESA study also found that women had higher total cholesterol levels and performed less weekly physical activity than men, but reported a higher average systolic blood pressure in women^15^. This latter finding may be attributable to the fact that the mean age of women enrolled in the study was 62 ± 10 years, which is older than participants in the Heart SCORE study. In our study, women were younger (mean age 43.7 ± 12.5 years) than in MESA, yet we still observed higher total cholesterol levels and fewer weekly minutes of vigorous activity, similar to what was observed in the other cohort studies. We also found that women were more likely than men to have ideal measures for ≥ 5 cardiovascular health and behavior categories. Using National Health and Nutrition Examination Survey (NHANES) data, in 2015–2016 ~ 22% of women met ideal criteria for ≥ 5 Life’s Simple 7 categories while only ~ 13% of men met this metric^4^.

A major finding from our study is the discovery of phenotypic heterogeneity among women and its implications for examining sex-based differences in ideal cardiovascular health. The concept of heterogeneity among patients with a common “phenotype” and the use of cluster analyses to define phenogroups has been described^19^. For example, heart failure and preserved ejection fraction (HFpEF) is recognized as a heterogeneous clinical syndrome, yet it is often considered as a single phenotype for the purposes of enrollment in trials or therapeutics. When cluster analysis and phenomapping was applied to a cohort of 397 patients with HFpEF, 3 distinct phenogroups emerged. These phenogroups differed in clinical characteristics as well as outcomes^19^. This methodology has been applied in other cohorts with HFpEF, dilated cardiomyopathy, and aortic stenosis undergoing transcatheter aortic valve replacement as well as for clinically relevant tests, including echocardiographic variables associated with heart failure and cardiopulmonary exercise testing^26–30^. In contrast to these studies, we elected to explore heterogeneity among women enrolled in the study as most sex-based comparisons in studies are done by considering women and men in aggregate. This allowed us to identify two phenogroups of women with different clinical profiles. Moreover, when we compared each of the phenogroups to men enrolled in the study, there were notable differences seen in the Life’s Simple 7 cardiovascular health and behavior categories that were not readily apparent from simple comparisons between women and men. These findings persisted even after adjusting for age, race and ethnicity, region, and affluence index, a marker of socioeconomic status.

Our finding of phenotypic heterogeneity in ideal cardiovascular health among women is supported by analyses from the Framingham Heart Study Offspring Cohort and NHANES. One report from the Framingham Heart Study Offspring Cohort study identified heterogeneity in longitudinal trends of Life’s Simple 7 Health Scores for women (and men) but did not explore these differences in the context of the individual factors that comprise the Life’s Simple 7 Health Score as our study did^11^. An analysis from NHANES revealed temporal differences in ideal cardiovascular health for non-Hispanic black and Mexican–American women as compared to non-Hispanic white women. In this study, sex-based differences were stratified by race and ethnicity as well as age. Although the study reported heterogeneity between the race and ethnic groups in Life’s Simple 7 Health Score and the 7 overall Health Score categories, it did not evaluate heterogeneity within each group of women^31^. A more recent study that included a larger sample from the NHANES cohort also described heterogeneity in ideal cardiovascular health among women by categorizing the percentage of women who scored ideal, intermediate, or poor for each of the Life’s Simple 7 categories but also did not explore this heterogeneity at a granular level. Similar to what we report, female sex was also identified as an independent factor associated with ideal cardiovascular health^32^.

The importance of identifying phenotypically different clusters of women with different levels of ideal cardiovascular health is underscored by studies that identified an association between higher Life’s Simple 7 Health Scores with lower rates of incident cardiovascular disease or as a predictor of better longer-term health outcomes^11,13,33^. Our cluster analysis unmasks two very different sub-populations of women that would otherwise not be identified in an observational study where women might be considered in aggregate. In our study, women assigned to cluster 1 had lower ideal scores for each of the Life’s Simple 7 categories and lower ideal cardiovascular health. Our analysis suggests that cluster 1 would be more likely to benefit from interventions to attain ideal cardiovascular health that would lower their longer-term risk of cardiovascular diseases compared to women in phenogroup 2. Thus, identifying phenogroups of women has clinical implications for long-term risk stratification and intervention.

A second finding in our study was that incorporating digital health device measured data instead of self-reported data affected the Healthy Weight and Activity scores for women in both phenogroups as well as men. This also has implications for assessing ideal cardiovascular health using the Life’s Simple 7 Health Score as a recent longitudinal study found that when Life’s Simple 7 was calculated on a 0–14 point scale, each 1 unit increase in the Health Score was associated with an estimated 12% lower risk of major adverse cardiovascular events^34^. In our study, participants under- and overreported their weight and overreported minutes of moderate activity while underreporting minutes of vigorous activity. This is not surprising as the Women’s Health Study found that while self-reported and measured weight had a high correlation (0.97), women both under- and overreported their weight^35^. Similarly, it’s also been shown that when comparing self-reported to objectively measured exercise using a digital device, women tended to overreport exercise minutes on the survey^36^.

In our study, the benefit of utilizing digital health device data was recognized most notably by women in phenogroup 1, the phenogroup with a higher burden of cardiovascular disease risk factors and established cardiovascular disease as compared to men. Using self-reported data, this phenogroup had a lower Health Score than men, but there was no difference between the groups when digital health data was used to calculate the Health Score. This suggests that digital health devices merit consideration for use in clinical studies that include participants with higher risk cardiovascular profiles. Among individuals with established cardiovascular disease, an analysis of 10 studies found that adherence to the use of digital health devices for exercise or activity monitoring ranged from 39.6 to 85.7%^37^. While there is variability in the rate of adherence, our study demonstrated the utility of incorporating digital health data in an assessment of ideal cardiovascular health for participants with high-risk cardiovascular profiles.

There are a number of limitations that could influence the generalizability of our findings. First, we enrolled far fewer men than women, which may have biased the findings in men. Second, we did not collect additional health information from the women, such as menopausal status, which would be an important consideration when examining cardiovascular disease health metrics and behaviors. It is also well recognized that women with pregnancy-related complications are at increased lifetime risk of cardiovascular disease compared with healthy controls^38,39^. As our study did not collect information on pregnancy or pregnancy-related complications and we did not have access to medical records to obtain this information, we were unable to examine this important determinant of women’s cardiovascular health. We also noted differences in dietary habits between the phenogroups with women who had lower ideal cardiovascular health avoiding prepackaged foods and eating away from home but reporting higher sodium intake than women with ideal cardiovascular health. The factors underlying this may be related to socioeconomic status, region, and/or awareness; however, further study is necessary to explain this finding. Also, the use of digital health devices in the study was optional by design. It is, therefore, possible that participants that contributed data from these devices were more motivated to achieve ideal health metrics in each of the Life’s Simple 7 categories. This may especially be true in women with cardiovascular disease where a 12-week pilot study of a mobile health device-based intervention resulted in improvements in BMI, waist circumference, and depressive symptoms^40^. Furthermore, as there were no differences in average daily step counts between the phenogroups of women and men, it is unlikely that there were differences in device use between the groups.

The concept of unrecognized phenotypic heterogeneity among participants in clinical trials achieves importance owing to the fact that it can bias interpretation of study outcome. This was shown in the My Research Legacy study cohort by demonstrating how resolving heterogeneity among women by cluster analysis affected interpretation of sex-based differences in ideal cardiovascular health metrics and behaviors. The study also underscores the value of digital health devices as a mechanism to provide robust unbiased data for the assessment of ideal cardiovascular health. Furthermore, the study illustrates the potential of applying precision medicine analytics to clarify heterogeneity and improve health in populations by identifying phenogroups that can be targeted selectively for health and lifestyle interventions. Thus, our findings suggest that a precision medicine approach to heterogeneity in clinical trial cohorts represents a novel mechanism into a more precise method to improve population health.

## Supplementary Information

Supplementary Information.

## References

[1] Leopold JA, Loscalzo J (2018). Emerging role of precision medicine in cardiovascular disease. Circ. Res..

[2] Antman EM, Loscalzo J (2016). Precision medicine in cardiology. Nat. Rev. Cardiol..

[3] Benjamin EJ (2017). Heart disease and stroke statistics-2017 update: A report from the American Heart Association. Circulation.

[4] Virani SS (2020). Heart disease and stroke statistics-2020 update: A report from the American Heart Association. Circulation.

[5] Nowbar AN, Gitto M, Howard JP, Francis DP, Al-Lamee R (2019). Mortality from ischemic heart disease. Circ. Cardiovasc. Qual. Outcomes.

[6] Lloyd-Jones DM (2010). Defining and setting national goals for cardiovascular health promotion and disease reduction: The American Heart Association's strategic Impact Goal through 2020 and beyond. Circulation.

[7] Angell SY (2020). The american heart association 2030 impact goal: A presidential advisory from the American Heart Association. Circulation.

[8] Sanchez E (2018). Life's simple 7: Vital but not easy. J. Am. Heart Assoc..

[9] Collins TC (2017). Ideal cardiovascular health and peripheral artery disease in African Americans: Results from the Jackson Heart Study. Prev. Med. Rep..

[10] Nayor M, Enserro DM, Vasan RS, Xanthakis V (2016). Cardiovascular health status and incidence of heart failure in the framingham offspring study. Circ. Heart Fail..

[11] Enserro DM, Vasan RS, Xanthakis V (2018). Twenty-year trends in the American Heart Association Cardiovascular Health Score and Impact on Subclinical and Clinical Cardiovascular Disease: The Framingham offspring study. J. Am. Heart Assoc..

[12] Spahillari A (2017). Ideal cardiovascular health, cardiovascular remodeling, and heart failure in blacks: The Jackson heart study. Circ. Heart Fail..

[13] Corlin L, Short MI, Vasan RS, Xanthakis V (2020). Association of the duration of ideal cardiovascular health through adulthood with cardiometabolic outcomes and mortality in the Framingham offspring study. JAMA Cardiol..

[14] Jankovic J (2016). Sex inequalities in cardiovascular health: A cross-sectional study. Eur. J. Public Health.

[15] Osibogun O, Ogunmoroti O, Tibuakuu M, Benson EM, Michos ED (2019). Sex differences in the association between ideal cardiovascular health and biomarkers of cardiovascular disease among adults in the United States: A cross-sectional analysis from the multiethnic study of atherosclerosis. BMJ Open.

[16] Leopold, J. A., Davis, R. B. & Antman, E. M. Data from digital health devices informs ideal cardiovascular health. *J. Pers. Med. ***11**, 189. 10.3390/jpm11030189 (2021). 10.3390/jpm11030189 PMC7998383 33801949

[17] Clarke P (2014). Cumulative exposure to neighborhood context: Consequences for health transitions over the adult life course. Res. Aging.

[18] Clarke, P. J. & Melendez, R. *National Neighborhood Data Archive (NaNDA): Neighborhood socioeconomic and demographic characteristics of census tracts, United States, 2000–2010 (V1). Inter-university Consortium for Political and Social Research*, https://www.openicpsr.org (2019).

[19] Shah SJ (2015). Phenomapping for novel classification of heart failure with preserved ejection fraction. Circulation.

[20] Ward JHJ (1963). Hierarchical grouping to optimize an objective function. J. Am. Stat. Assoc..

[21] Escofier B, Pages J (1994). Multiple factor analysis (AFMULT package). Comput. Stat. Data Anal..

[22] Gower JC (1971). A general coefficient of similarity and some of its properties. Biometrics.

[23] Cash RE, Crowe RP, Bower JK, Foraker RE, Panchal AR (2019). Differences in cardiovascular health metrics in emergency medical technicians compared to paramedics: A cross-sectional study of emergency medical services professionals. Prehosp. Disaster Med..

[24] Bambs C (2011). Low prevalence of "ideal cardiovascular health" in a community-based population: The heart strategies concentrating on risk evaluation (Heart SCORE) study. Circulation.

[25] Erqou S (2018). Ideal cardiovascular health metrics in couples: A community-based study. J. Am. Heart Assoc..

[26] Segar MW (2020). Phenomapping of patients with heart failure with preserved ejection fraction using machine learning-based unsupervised cluster analysis. Eur. J. Heart Fail..

[27] Verdonschot JAJ (2021). Phenotypic clustering of dilated cardiomyopathy patients highlights important pathophysiological differences. Eur. Heart J..

[28] Abdul Ghffar Y (2020). Usefulness of semisupervised machine-learning-based phenogrouping to improve risk assessment for patients undergoing transcatheter aortic valve implantation. Am. J. Cardiol..

[29] Mishra RK (2020). Association of machine learning-derived phenogroupings of echocardiographic variables with heart failure in stable coronary artery disease: The heart and soul study. J. Am Soc Echocardiogr.

[30] Oldham WM (2018). Network analysis to risk stratify patients with exercise intolerance. Circ. Res..

[31] Pool LR, Ning H, Lloyd-Jones DM, Allen NB (2017). Trends in racial/ethnic disparities in cardiovascular health among US adults from 1999–2012. J. Am. Heart Assoc..

[32] Egan BM (2020). Sociodemographic determinants of life's simple 7: Implications for achieving cardiovascular health and health equity goals. Ethn. Dis..

[33] Xanthakis V (2014). Ideal cardiovascular health: Associations with biomarkers and subclinical disease and impact on incidence of cardiovascular disease in the Framingham Offspring Study. Circulation.

[34] Nguyen ATH (2021). Usefulness of the American Heart Association's ideal cardiovascular health measure to predict long-term major adverse cardiovascular events (from the heart SCORE study). Am. J. Cardiol..

[35] Luo J (2019). Accuracy of self-reported weight in the Women's Health Initiative. Public Health Nutr..

[36] Hartley S (2015). A comparison of self-reported and objective physical activity measures in Young Australian Women. JMIR Public Health Surveill..

[37] Marin TS (2019). Examining adherence to activity monitoring devices to improve physical activity in adults with cardiovascular disease: A systematic review. Eur. J. Prev. Cardiol..

[38] Cusimano MC, Pudwell J, Roddy M, Cho CK, Smith GN (2014). The maternal health clinic: An initiative for cardiovascular risk identification in women with pregnancy-related complications. Am. J. Obstet. Gynecol..

[39] Siu SC (2021). Long-term cardiovascular outcomes after pregnancy in women with heart disease. J. Am. Heart Assoc..

[40] Sengupta A, Beckie T, Dutta K, Dey A, Chellappan S (2020). A mobile health intervention system for women with coronary heart disease: Usability study. JMIR Form. Res..

