# Supplementary material for: A precision medicine approach to sex-based differences in ideal cardiovascular health
Source: Sci Rep. 2021 Jul 21;11:14848. doi: 10.1038/s41598-021-93966-3 (PMC8295282; doi:10.1038/s41598-021-93966-3)
Supplement: Supplementary file 1 — Supplementary Information. [file 41598_2021_93966_MOESM1_ESM.pdf]

SUPPLEMENTARY DATA

A precision medicine approach to sex-based differences in ideal cardiovascular health

Jane A. Leopold, MD[1] and Elliott M. Antman, MD[1]

[1]Division of Cardiovascular Medicine, Brigham and Women's Hospital,

Harvard Medical School

Supplemental Table 1. Self-reported Life's Simple 7 data

	REPORTED IN LIFE'S SIMPLE 7 SURVEY					
	**Cluster 1 (n=614)**	**Cluster 2 (n=637)**	**Men (n=310)**	**P value Cluster 1 vs. 2**	**P value Cluster 1 vs. Men**	**P value Cluster 2 vs. Men**
Age (yrs)	47.4 ± 10.9	40.0 ± 12.8	46.3± 15.0	<0.01	0.19	<0.01
Race and Ethnicity				0.54	<0.01	0.23
(no.)	9	16	17			
Asian	22	27	11			
Black	24	30	14			
Hispanic	534	543	260			
White	25	21	8			
Other						
Affluence index	0.40 ± 0.14	0.43 ± 0.15	0.44 ± 0.16	<0.01	<0.01	0.50
Region (no.)				<0.01	0.20	<0.02
Northeast	98	92	37			
South	273	214	135			
Midwest	145	158	75			
West	98	173	63			
Diagnosed with Cardiovascular Disease (%)	54.2	16.6	41.0	<0.01	<0.01	<0.01
Diabetes mellitus (%)	16.3	4.4	10.7	<0.01	<0.03	<0.01
Hypertension (%)	73.1	22.3	60.3	<0.01	<0.01	<0.01
Hypercholesterolemia (%)	78.2	28.9	58.8	<0.01	<0.01	<0.01
Medications (%)						
Diabetes mellitus	14.2	3.6	8.7	<0.01	<0.02	<0.01
Hypertension	50.2	11.6	40.3	<0.01	<0.01	<0.01
	31.9	5.0	30.0	<0.01	0.55	<0.01
Hypercholesterolemia						
Smoking status (%)				<0.01	<0.05	0.15
Current	8.6	6.1	5.2			
Quit < 12 months	4.7	3.0	3.6			
Quit ≥ 12 months	28.7	18.4	24.5			
Never	58.0	72.5	66.8			
Weight (kg)	87.7 ± 24.4	75.8 ± 21.8	94.6 ± 23.8	<0.01	<0.01	<0.01
BMI (kg/m^2)	32.5 ± 9.0	27.8 ± 7.5	29.4 ± 6.5	<0.01	<0.01	<0.01

Systolic blood pressure (mmHg)*	121.1 ± 14.0	113.2 ± 10.1	122.2 ± 11.9	<0.01	0.25	<0.01
Diastolic blood pressure (mmHg)*	75.4 ± 9.4	70.4 ± 7.2	75.2 ± 8.1	<0.01	0.77	<0.01
Total cholesterol (mg/dL)*	196.1 ± 29.0	185.0 ± 25.4	181.4 ± 33.7	<0.01	<0.01	<0.01
Blood glucose (mg/dL)*	101.5 ± 19.7	94.9 ± 15.9	103.7 ± 17.3	<0.01	0.10	<0.01
**DIET**						
Vegetables/day (cups)	1.6 ± 1.2	2.2 ± 1.4	1.7 ± 1.2	<0.01	0.28	<0.01
Fruit/day (cups)	1.5 ± 1.1	1.3 ± 1.0	1.2 ± 1.1	<0.01	0.32	<0.01
Fish (servings/week)	0.9 ± 1.0	0.8 ± 1.0	1.1 ± 1.1	<0.01	<0.01	<0.05
Whole grains (servings/day)	1.7 ± 1.2	1.5 ± 1.1	1.6 ± 1.2	<0.01	<0.02	0.33
Sugar-sweetened beverages (servings/week)	2.2 ± 3.2	2.7 ± 3.6	2.4 ± 3.3	<0.02	0.26	0.34
Avoid prepackaged foods (%)	59.3	41.9	52.3	<0.01	<0.01	<0.01
Avoid eating out (%)	36.4	41.1	34.8	<0.01	<0.01	<0.01
Avoid salt at home (%)	42.8	76.5	56.8	<0.04	0.41	0.37
**EXERCISE**						
Moderate exercise (min/week)	179.8 ± 204.5	222.2 ± 224.1	214.4 ± 212.4	<0.01	<0.02	0.61
Vigorous exercise (min/week)	36.3 ± 82.0	85.5 ± 128.8	91.3 ± 135.1	<0.01	<0.01	0.52
**LIFE'S SIMPLE 7 SCORES**						
Smoking score (%)<br>Poor<br>Intermediate<br>Ideal	 8.6<br>4.7<br>86.6	 6.1<br>3.0<br>90.9	 5.2<br>3.6<br>91.3	<0.02	<0.04	0.82
Physical activity score(%)<br>Poor<br>Intermediate<br>Ideal	 2.8<br>47.1<br>50.1	 1.3<br>30.1<br>68.6	 1.6<br>34.5<br>63.9	<0.01	<0.01	0.14
Healthy diet score (%)<br>Poor	 57.3	 32.0	 43.2	<0.01	<0.01	<0.01

Intermediate<br>Ideal	37.6<br>5.1	55.9<br>12.1	47.7<br>9.0			
Healthy weight score (%)<br>Poor<br>Intermediate<br>Ideal	 <br>55.4<br>22.3<br>22.3	 <br>32.1<br>22.6<br>46.3	 <br>39.4<br>31.3<br>29.4	<0.01	<0.01	<0.01
Blood glucose score (%)<br>Poor<br>Intermediate<br>Ideal	 5.9<br>38.1<br>56.0	 2.1<br>20.7<br>77.2	 3.6<br>61.9<br>34.5	<0.01	<0.01	<0.01
Cholesterol score (%)<br>Poor<br>Intermediate<br>Ideal	 3.9<br>73.1<br>23.0	 1.6<br>26.5<br>71.9	 1.9<br>51.6<br>46.5	<0.01	<0.01	<0.01
Blood pressure score (%)<br>Poor<br>Intermediate<br>Ideal	 <br>9.8<br>68.2<br>22.0	 <br>2.0<br>30.8<br>67.2	 <br>7.4<br>66.1<br>26.5	<0.01	<0.08	<0.01
LS7 Health Score	5.9 ± 1.3	7.6 ± 1.3	6.4 ± 1.4	<0.01	<0.01	<0.01

**Contains data imputed from Life's Simple 7*
Categorical variables are analyzed by Chi-Square test
Continuous variables are analyzed by t-test
Non-parametric variables were analyzed by Wilcoxon rank-sum test

Supplemental Table 2. Digital health device weight and exercise data

	Cluster 1 (n=132)	Cluster 2 (n=103)	Men (n=57)	P value Cluster 1 vs 2	P value Cluster 1 vs. Men	P value Cluster 2 vs. Men
Weight (kg)	83.2 ± 22.1	73.9 ± 18.1	88.2 ± 18.2	<0.01	0.14	<0.01
BMI (kg/m^2)	30.4 ± 7.8	27.0 ± 6.4	27.4 ± 4.9	<0.01	<0.01	0.72
Healthy Weight Score (%)<br>Poor<br>Intermediate<br>Ideal	 <br>46.2<br>25.8<br>28.0	 <br>26.2<br>29.1<br>44.7	 <br>24.6<br>42.1<br>33.3	<0.01	<0.02	0.41
	**Cluster 1 (n=144)**	**Cluster 2 (n=137)**	**Men (n=74)**	**P value Cluster 1 vs 2**	**P value Cluster 1 vs Men**	**P value Cluster 2 vs Men**
Moderate exercise (min/week)	118.6 ± 145.1	139.6 ± 185.1	159.9 ± 206.7	0.29	<0.09	0.47
Vigorous exercise (min/week)	137.3 ± 209.6	159.7 ± 196.4	226.1 ± 303.0	0.36	<0.02	<0.06
Physical Activity Score (%)<br>Poor<br>Intermediate<br>Ideal	 <br>0.0<br>46.5<br>53.5	 <br>0.0<br>35.8<br>64.2	 <br>0.0<br>32.4<br>67.6	<0.07	<0.05	0.63
	**Cluster 1 (n=122)**	**Cluster 2 (n=97)**	**Men (n=53)**	**P value Cluster 1 vs 2**	**P value Cluster 1 vs Men**	**P value Cluster 2 vs Men**
LS7 Health Score	6.4 ± 1.2	7.9 ± 1.1	6.8 ± 1.2	<0.01	0.19	<0.01

390 individuals that registered devices and provided data: 307 women and 83 men. A total of 292 individuals had linked digital scales to their devices and provided weight data: 235 women (132 in cluster 1 and 103 in cluster 2) and 57 men. A total of 355 individuals contributed exercise data: 281 women (144 in cluster 1 and 137 in cluster 2) and 74 men.

Continuous variables are analyzed by t-test
Non-parametric variables were analyzed by Wilcoxon rank-sum test

LEGEND

Supplemental Figure 1. Generalizability of the My Research Legacy sample. The My Research Legacy study enrolled a heterogeneous group of participants that included individuals from A) different age groups and B) socioeconomic status demonstrated by the affluence index. C) The study also enrolled participants from all 50 states. State names are represented by standard 2 letter name code.

Supplemental Figure 2. Determinants of an ideal cardiovascular health score. Multivariable logistic regression identified determinants of having an ideal cardiovascular health score. The odds ratio and 95% confidence interval are shown.

Supplemental Figure 3. Contributors to variability in the first two dimensions. A factor analysis of mixed data was performed to reduce dimensionality of the data and to examine similarities between participants. This revealed that the first two dimensions accounted for 19.8% of the variability among participants. A) The contributors to variability for the first dimension (Dim-1) and B) the second dimension (Dim2) are shown. The dashed red line represents the expected contribution if all factors contributed equally to the variability. MedBP, medications for hypertension; HTN, hypertension; SBP, systolic blood pressure; Hchol, hypercholesterolemia; BMI, body mass index; DBP, diastolic blood pressure; MedChol, medications for hypercholesterolemia; Age, age at enrollment; DM, diabetes mellitus; Glucose, blood glucose level; VigEx, weekly minutes of vigorous exercise; CVD, cardiovascular diseases; Smoking, current or former tobacco user; Veggies, cups of vegetables per day; Chol, blood cholesterol level; Fruit, cups of fruit per day; Grains, servings per day of whole grains; ModEx, weekly minutes of moderate exercise; Prepack Food, avoids prepackaged foods; Region, region of country; Salt, avoids added salt; Ethnicity, race and ethnicity; DigDev, registered a digital health device; SugarDrink, ounces per week of sugar-sweetened beverages; Fish, servings of fish per week; EatOut, avoids eating out.

Supplemental Figure 4. Silhouette width to determine the optimal number of clusters. In order to determine the optimal number of clusters, the average silhouette width was calculated using the cluster package in R (version 3.6.2). A silhouette coefficient of >0.5 is considered reliable.

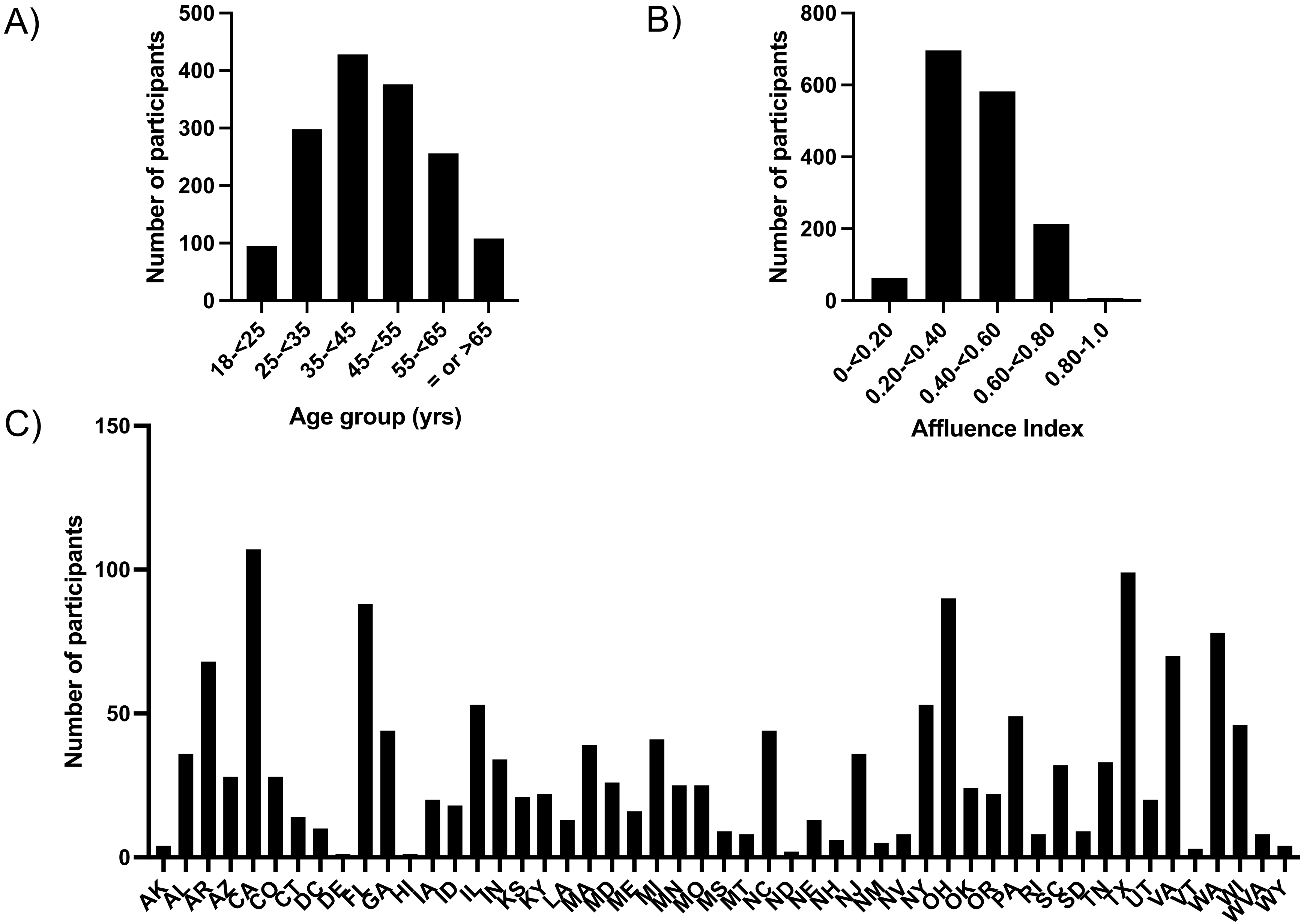

Supplemental Fig. 1. Generalizability of the My Research Legacy sample.

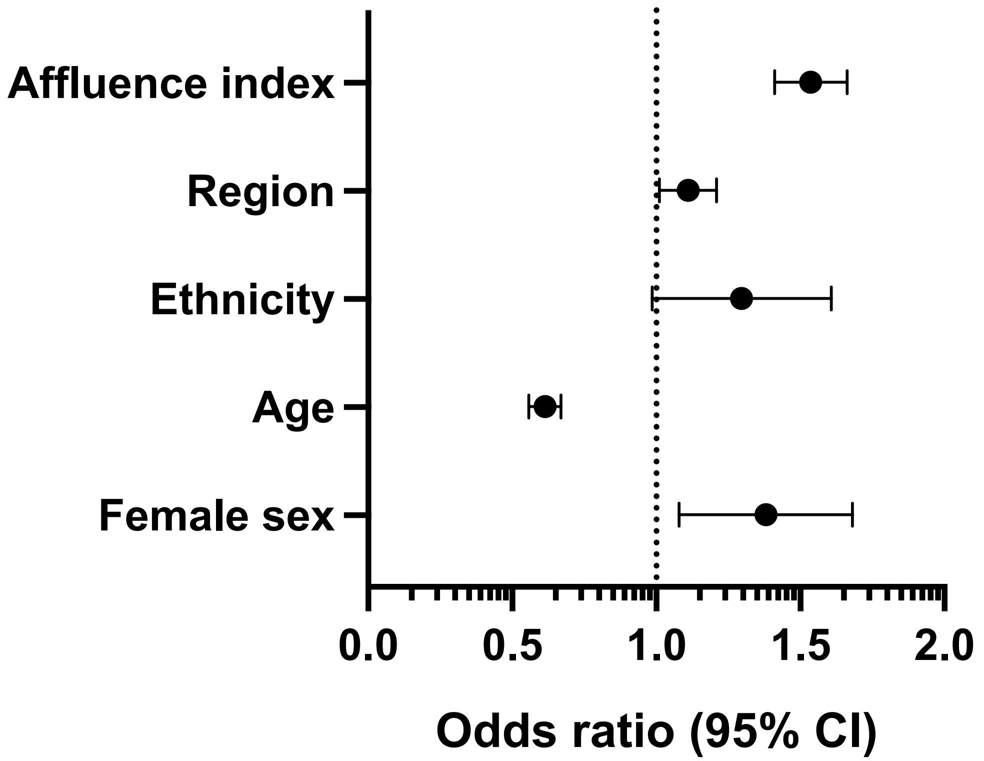

Supplemental Fig. 2. Determinants of an ideal cardiovascular health score.

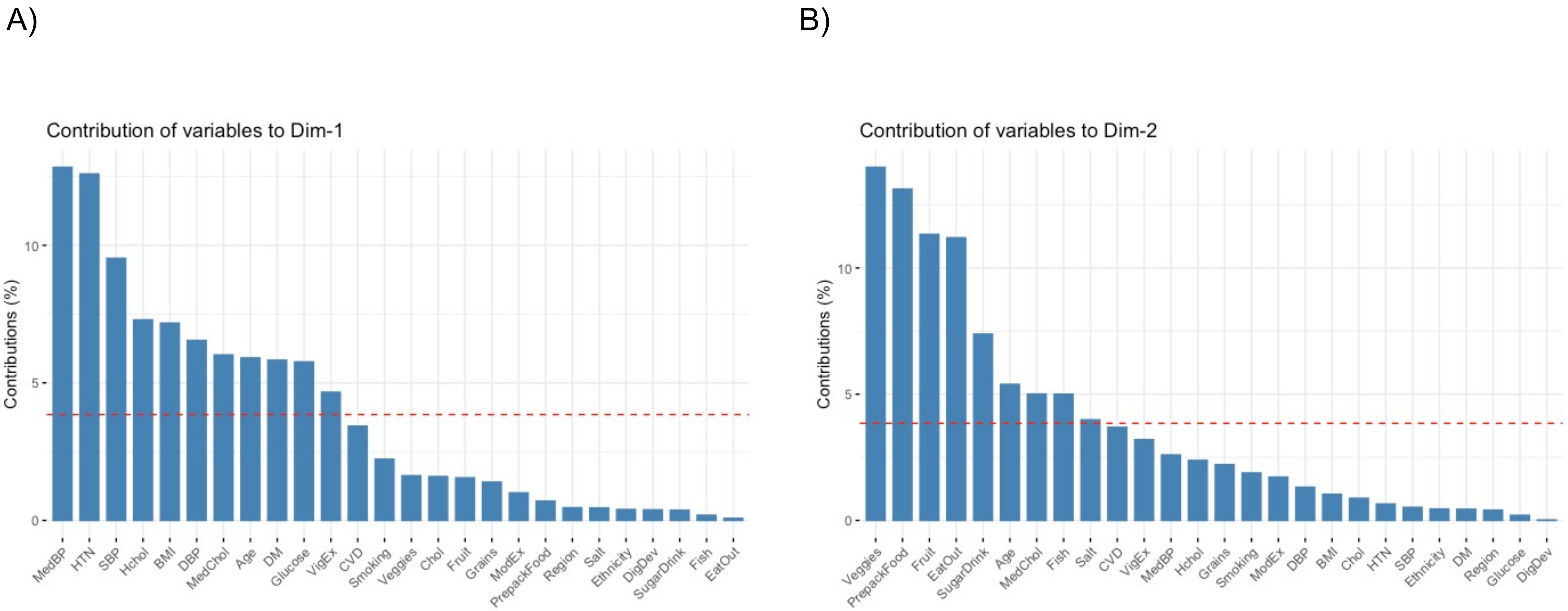

Supplemental Fig. 3. Contributors to variability in the first two dimensions

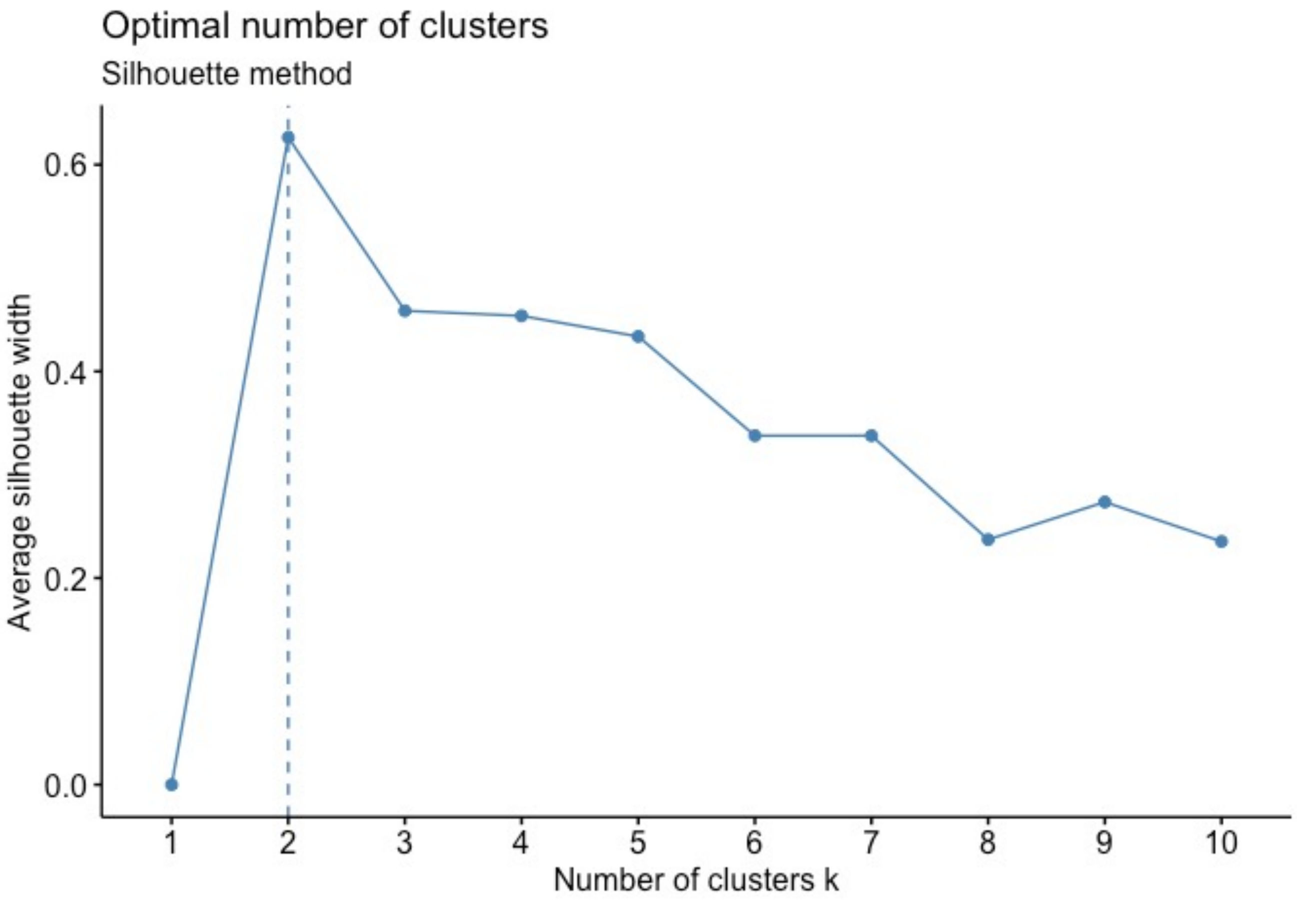

Supplemental Fig. 4. Silhouette width to determine the optimal number of clusters
